# The Book World of Medicine and Science

**Published:** 1897-10-02

**Authors:** 


### THE BOOK WORLD OF MEDICINE AND SCIENCE.

System of Diseases of the Eye. By American, British, Dutch, French, and Spanish Authors. Edited by William F. Norris, A.M., M.D., and Charles A. Oliver, A.M., M.D., of Philadelphia, P.A., U.S.A. In four volumes. Volume I.—Embryology, Anatomy, and Physiology of the Eye. Pp. 670, with 23 full-page plates and 362 text illustrations. (London and Philadelphia: J. B. Lippincott and Co.) 1897. Price of the complete work by subscription, 20 dols.

The editors of this handsome volume remark in their preface that no system of eye diseases has hitherto appeared in the English language. Whether this is, as they obviously consider, wholly a disadvantage is perhaps questionable, but there can be no doubt that they have set about the filling of the gap in a most thorough and scientific spirit, and bid fair to produce a work which, when complete, will far excel any previous attempts in the same direction. They have sought their contributors with wise catholicity, and will have the co-operation of many, among whom we may mention Snellen, Laudolt, Schnabel, Nuel, Priestley Smith, and Haab, who are unquestionably the most eminent living authorities on the subjects of which they treat. The volume under notice opens with a careful and lucid account of the development of the eye, to which a melancholy interest attaches as the last work of the late distinguished Professor John A. Ryder. This is followed by Professor Dwight's article on the "Anatomy of the Orbit and the Appendages of the Eye." This is mainly topographical, and wisely accentuates the points of most importance in ophthalmic surgery; we can give it no higher praise than to say that it is worthy of the reputation of its author as the foremost among American anatomists. The succeeding memoir, that of Professor Frank Baker, treats of the anatomy of the eyeball; it is extremely and minutely complete, but we cannot help thinking that 108 pages is rather too much space to allot to this subject in a medical book. The next article is of even greater length—157 pages—but considering the amount of new and in part controversial matter that has to be dealt with, we cannot grudge Professor Piersol his allowance. His subject is the microscopical anatomy of the eyeball, and its treatment gives every evidence both of wide reading and accurate judgment. This is unquestionably the best article in the book, and the part relating to the histology of the retina is probably as good an account as will be found in any language. Dr. Alexander Hill's description of the intracranial portion of the visual apparatus is a little disappointing, some of the information it contains being rather out of date; but we have nothing but praise for Messrs. Lang and Treacher Collins' article on "Congenital Malformations and Abnormalities of the Eye." Professor Edward Jackson treats of the subject on which he is a recognised authority, ocular dioptrics, his account of which is clear, simple, and but little burdened with mathematical formulæ. Professor Cattell, the well-known New York psychologist, gives a description of the perception of light, which, though extremely interesting, seems a little unnecessary, and the same criticism applies, though in a less degree, to Dr. Brodhun's memoir on binocular vision, in which the medical man is afforded the opportunity of meeting again that old enemy of his physiological days, the horopter. Normal colour perception is treated by Professor William Thomson and Dr. Carl Weiland, whose article is both reasonable and readable. They prefer the Young-Helmholtz theory of colour vision to that of Hering, which is, perhaps, rather more popular in England; their brief account of the probable evolution of colour perception is speculative, but very interesting. The last article in the book is on "Photo-Chemistry of the Retina," by Dr. Carl Mays, who, as a pupil and assistant of Kühne, is well qualified to speak on this fascinating subject, which has not yet, we fear, assumed practical clinical importance. The whole volume has both the advantages and drawbacks of the encyclopædic method. On the one hand, if we may borrow a metaphor from the subject, to study questions from different aspects gives them a stereoscopic solidity, on the other there is frequently overlapping and occasionally contradiction. Thus two authors give careful descriptions of the canal of Schlemm, while a third denies, almost with scorn, its very existence. There also seems to be a divergence of opinion as to the spelling choroid or chorioid, in which we regret to see the latter appears to be gaining the day. The book can, however, be unhesitatingly declared essential for all those who desire to make the eye their study; it is superbly produced, and the illustrations are wholly admirable.

The A B C of the X Rays. By William H. Meadowcroft, author of "The A B C of Electricity." (London: Simpkin, Marshall, Hamilton, Kent, and Co.) 1897. Crown 8vo., pp. 190, 37 illustrations. 4s.

A very practical and useful little book, containing all that is necessary to enable anyone with no previous knowledge of electricity to select and intelligently work the necessary apparatus for taking X ray pictures. In a short introduction the author gives a general outline of Röntgen's great discovery, in the course of which he corrects certain popular misconceptions that have arisen as to some of the phenomena and the method of their utilisation. Then follows a description of the apparatus and materials that experience has shown to be most useful. Everything is described in the simplest language, which, although popular, is not misleading. Mr. Meadowcroft has managed to give a wonderfully connected story of a subject which has been, and is still, beyond the understanding of anyone. Chapter III., on electrical induction, gives a very clear account of a subject which all who propose to work at the Röntgen ray should have at their fingers' end, and the knowledge gained in this chapter is put to practical use in Chapter IV., which considers the construction of the induction coil. Perhaps in a volume of this kind, the rather full description that is given to high frequency apparatus and to statical machines might have been reduced, for both are difficult to manage, and so far have not come into general use for exciting Crookes' tubes, although good results have undoubtedly been obtained by their employment. We are sorry to find that the credit of introducing the now universal "focus tube" is given, not to Mr. Herbert Jackson, who, by-the-bye, was using this type of tube even before Röntgen's memorable discovery, but to a distinguished American investigator. Doubtless Mr. Meadowcroft has been misinformed upon this subject. In the chapter on manipulation some practical hints are given, together with very clear instructions as to utilising current from street mains, which will be found useful. We have no hesitation in confidently recommending the book to all beginners, and it will ensure a desire for a deeper insight into one of the most wonderful and so far inexplicable of the many electrical discoveries of the century.

